# Survey of Radon Concentrations in the University of Granada in Southern Spain

**DOI:** 10.3390/ijerph18062885

**Published:** 2021-03-11

**Authors:** Irene Calvente, María Isabel Núñez, Rachid Chahboun Karimi, Juan Villalba-Moreno

**Affiliations:** 1 Department of Radiology and Physical Medicine, School of Medicine, University of Granada, 18016 Granada, Spain; irenejaramal@gmail.com (I.C.); jvillal@ugr.es (J.V.-M.); 2 Research Support Unit, Biosanitary Institute of Granada (ibs.GRANADA), San Cecilio University Hospital of Granada, 18016 Granada, Spain; 3 Biopathology and Regenerative Medicine Institute (IBIMER), University of Granada, 18016 Granada, Spain; 4 Radiological Protection Service, University of Granada, 18010 Granada, Spain; rachid@ugr.es

**Keywords:** radon health effects, indoor radon levels, radon exposure, ventilation

## Abstract

The objective of this pilot study was to gather and analyze data on radon concentrations in workplaces in three buildings of Granada University (Southern Spain) constructed in different centuries. All measurements were made at basement or ground floor level under normal use conditions except for one space (mineral store), in which measurements were compared between the door closed and open. Measurements were conducted during different time periods between October 2013 and March 2019 with a Radon-Scout PLUS portable Radonmonitor. The duration of continuous recordings at different sites ranged between 42 and 1104 h. Mean accumulated radon concentrations ranged between 12 and 95 Bq/m^3^, below the maximal level of 300 Bq/m^3^ set by the World Health Organization (WHO). Relatively high values were recorded in the oldest building (15th century), which was also poorly ventilated. Ventilation appeared to be an important factor in reducing radon levels, especially in areas less exposed to radon, such as Southern Spain.

## 1. Introduction

Radon is a major component of the ionizing radiation dose received by the general population, and there is no zero-risk level [1]. Since 1998, radon and disintegration products have been considered human carcinogens (group 1) by the International Agency for Research on Cancer (IARC) of the World Health Organization (WHO) due to their known effects on the lung. Exposure to indoor radon is estimated to be the second cause of lung cancer after tobacco smoking [1,2,3,4].

Radon (Rn-222) derives from the radium (Ra-226) present in soils (depending on the rock type), water (when from underground streams in which the gas is dissolved), or construction materials (e.g., ceramic bricks, concrete, plaster, cement, fly ash) [5]. This radioactive gas tends to concentrate in enclosed spaces such as underground mines, workplaces, and dwellings. Its concentration in buildings largely depends on the uranium content of the earth’s crust beneath them, their construction materials, age, and ventilation [1,5,6]. It has been estimated that materials used in construction are responsible for 20% of the total radon recorded in rooms, making a contribution of 5–20 Becquerel/m^3^ (Bq/m^3^) [5,7]. The half-life (3.82 days) of radon is sufficiently long for the gas to spread from its source and accumulate in enclosed indoor spaces, posing a potential health threat [8,9].

Indoor radon surveys carried out in a sample of 5600 dwellings in Spain between 1990 and 2005, performing measurements in each dwelling for three months, reported a mean indoor radon exposure of 90 Bq/m^3^ with a geometric mean of 45 Bq/m^3^ [10,11]. A pilot study of indoor radon exposure in workplaces in different Spanish regions found radon concentrations higher than 300 Bq/m^3^ in 46% of workplaces in Galicia (*n* = 58) and in 10.6% of those in Madrid (*n* = 11). Out of 640 workers evaluated at their workplaces, 19% were exposed to radon concentrations higher than 300 Bq/m^3^ and 6.3% to concentrations higher than 500 Bq/m^3^ [12].

Epidemiological studies have associated long-term exposure to high concentrations of radon in workplaces and homes with a higher risk of developing lung cancer [1,11,13], and this risk was reported to depend on the indoor concentration of radon, the exposure time, and the degree of building ventilation [14,15,16]. In addition, a recent epidemiologic review on residential radon exposure and its impact on lung cancer risk found that, while tobacco is the main risk factor for lung cancer, residential radon is the first cause in never-smokers and the second in ever-smokers [17]. A pooled case-control study that only included never-smokers (523 cases and 892 controls) evidenced an association of residential radon exposure with the subsequent development of lung cancer. An odds ratio of 1.73 (95% CI: 1.27–2.35) was observed for individuals exposed to ≥200 Bq/m^3^ compared with those exposed to ≤100 Bq/m^3^. These authors described residential radon as a clear risk factor for lung cancer in never-smokers [18]. In addition, pooled studies in Europe [4], China [19], and North America [20] found that a rise of 100 Bq/m^3^ in the mean indoor concentration increases the likelihood of developing lung cancer by 10% [6].

Although radon has always been present in the environment we breathe, its concentration in interior spaces has significantly risen over recent years due to the increasing construction of buildings with improved thermal insulation and less ventilation. These changes increase energy saving but can lead to dangerous radon concentration levels, especially in poorly ventilated areas and those in closer contact with land, the main natural source of this gas, such as basements, semi-basements, and garages [21].

This pilot study was designed to evaluate radon levels in certain workplaces (where workers spend >8 h/day) within three university buildings in Granada, Southern Spain, one from the 15th century, one from the 20th century, and one from the 21st century, and to investigate factors associated with these levels. The aim was to examine the cumulative concentrations of radon in the air that may potentially be inhaled by the workers.

## 2. Materials and Methods 

Radon measurements were made in the three buildings under study during different time periods between October 2013 and March 2019 using a Radon-Scout PLUS portable radon monitor (SARAD GmbH, Dresden, Germany, serial number 390, purchased in 2010) calibrated at Cantabria University in May 2015. This instrument detects Rn-222 in ambient air based on a continuous cycle of measurements. An ionization chamber in the device stores data on concentrations of radon activity and on temperature, relative humidity, and atmospheric pressure at the time of measurement. All data are stored in an internal memory with a capacity for up to 2047 records. Measurement periods can be set from 1 to 255 min. It operates autonomously, with no requirement for membrane pumps, external power supply, or other mechanisms, facilitating its utilization at home or in the workplace. The device offers continuous data recording for at least four months. The calibration of the radon monitor was checked before measurements were made. 

In the present survey, the duration of continuous recordings at the different sites ranged between 42 h (<2 days) and 1104 h (46 days). Intervals established for the measurements were two or three hours, because intervals of less than one hour showed a high percentage error. All measurements were made at basement or ground floor level, with the detector placed at 1.20–2 m above floor level at a site unaffected by air currents or other types of interference. Detector parameters were as follows: measurement range, 0–10 MBq/cm^3^; response time, 120 min; relative humidity, 0 to 100%; temperature, −20 to 40 °C; pressure, 800 to 1200 mbar; and uncertainty, 1 to 2 Bq/m^3^. Further technical data are exhibited in Table 1. Measurements were made during the winter, when these buildings are more likely to remain closed to the exterior (e.g., windows shut), with the exception of the Porter’s Office of the School of Computer Sciences, which was measured in the spring and showed the lowest exposure levels.

### 2.1. Study Areas

The survey was conducted in seven areas in each of three university buildings, which were constructed in the 15th century (Royal Hospital), the 20th century (School of Sciences), and the 21st century (School of Computer Sciences). The Royal Hospital was constructed with stone (sedimentary rocks) and the other buildings with concrete.

### 2.2. Statistical Analysis 

Descriptive analysis of measurements was performed, computing arithmetic means with standard deviation (SD) and median values. Microsoft Excel 2010 was used for data analyses and Microsoft Photoshop Cs6 for graphics.

## 3. Results 

In this study of three university buildings in Southern Spain, radon levels were within the maximum limits established by the WHO. Levels were higher in the stone building from the 15th century than in the two concrete buildings constructed in the 20th and the 21st centuries, respectively. Table 2 exhibits the measurements obtained in each building, including mean (±SD) radon activity concentration, measurement duration, temperature, humidity, and pressure. Mean concentrations in the buildings ranged from 12 to 95 Bq/m^3^, with an overall mean of 43 Bq/m^3^. The highest mean value, (95 ± 46) Bq/m^3^, was recorded in the reading/study room of the oldest building (Royal Hospital).

In the School of Sciences, the four areas measured were in the basement, where the ventilation of each area was only provided by the door. With the doors closed, mean radon concentrations in the central laboratory (70 ± 59) Bq/m^3^ were 1.9-fold higher than in the warehouse (37 ± 21) Bq/m^3^, 1.8-fold higher than in the maintenance room (40 ± 27) Bq/m^3^, and 2-fold higher than in the documentation-archive room (32 ± 21) Bq/m^3^. This is in part because the detector in the central laboratory is immediately in front of the door to the room containing uranium.

**Figure 1:**
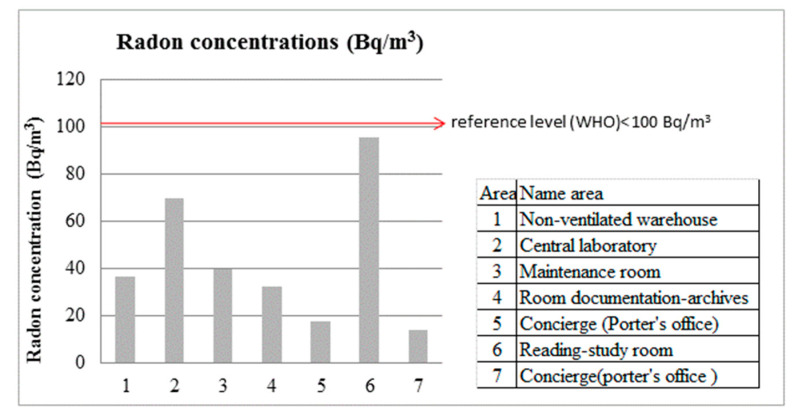
Mean indoor air radon concentrations at University of Granada (Southern Spain).

**Figure 2:**
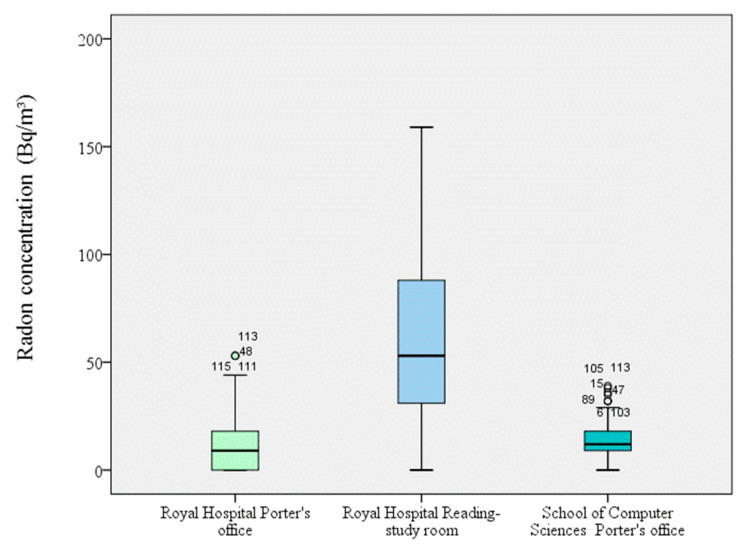
Radon in indoor air in 15th century (Royal Hospital) and 21st century (School Computer Sciences) buildings.

Among the areas measured on the ground floor in the three university buildings, radon concentrations were more than five-fold higher in the reading/study room of the Royal Hospital (95 ± 46) Bq/m^3^ than in the Porter’s offices in the same building (17 ± 17) Bq/m^3^ and the School of Computer Sciences (14 ± 8) Bq/m^3^ (Figure 1). Overall, radon concentrations were around two-fold higher in areas located in the basement (School of Sciences) than in those on the ground floor (Royal Hospital and School of Computer Sciences). It has previously been reported that concentrations of this gas are almost two-fold higher on the second floor of buildings than on lower floors [6]. Mean radon concentrations were higher in the 15th century building than in the more modern constructions, although this may also be influenced by differences in their ventilation (Figure 2). Lee et al. found no statistically significant correlation between radon concentrations and year of construction, although European reference levels differ between new buildings (200 Bq/m^3^) and existing buildings (400 Bq/m^3^) [9,22].

### Decontamination by Ventilation

**Figure 3:**
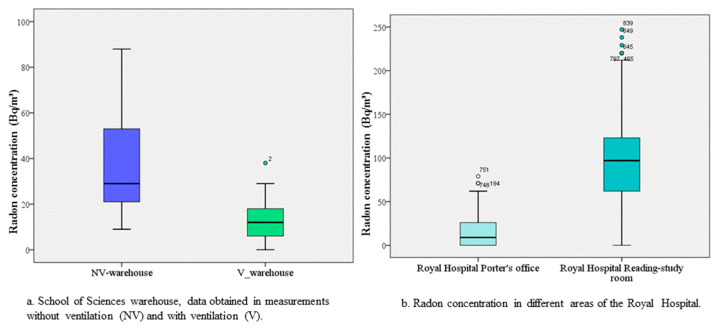
(**a**,**b**) Comparison of radon concentrations as a function of the presence or the absence of ventilation.

In the warehouse of the School of Sciences, the mean radon concentration was (37 ± 21) Bq/m^3^ with the door closed versus (12 ± 8) Bq/m^3^ with the door open (for 62 h), highlighting the importance of ventilation to reduce indoor radon exposure (Figure 3). Lee et al. observed a 1.7-fold reduction in mean levels when a room was ventilated (with open windows) for 2–3 h and reported a statistically significant correlation between radon levels and ventilation duration [9]. However, Dieguez-Elizondo et al. concluded that natural ventilation alone is not sufficient to keep radon concentrations below acceptable limits and that forced ventilation is required, although natural ventilation was considered adequate by Garcia-Talavera et al. when radon concentrations were below the WHO limit of 100 Bp/m^3^ [6,14]. Figure 3 shows that indoor radon concentrations were higher in non-ventilated versus ventilated areas in the present study.

## 4. Discussion

There has been little research on indoor radon concentrations in Spanish workplaces. No data are available on radon concentrations in workplaces in the University of Granada, prompting this pilot study in three different building of the institution. Mean accumulated radon concentrations detected in this study ranged between 12 and 95 Bq/m^3^, below the maximal level set by the WHO. The highest radon concentrations were found in the Royal Hospital, an old building made of stone, in agreement with previous findings on the influence of stone-based interior and exterior building materials on indoor radon concentrations [23]. The lowest indoor radon values were observed in ventilated areas, regardless of the age of the building.

Among the seven areas investigated in this study, the concentration of this radioactive gas ranged between 0–20 Bq/m^3^ in two areas and between 21 and 60 Bq/m^3^ in three areas and was above 61 Bq/m^3^ in the remaining two (Figure 1). The wide deviation in results can be attributed to various factors, including differences between day and night in these long-term measurements, varied ventilation conditions (opened/closed doors), and variations in pressure and temperature conditions. Potentially influential factors not considered in this pilot study include the meteorological conditions, which can affect the emission of radon by the land, an especially important radon source for ground floor areas such as those studied here. Further limitations of this preliminary study include the lack of data for each building during different seasons of the year. 

**Figure 4:**
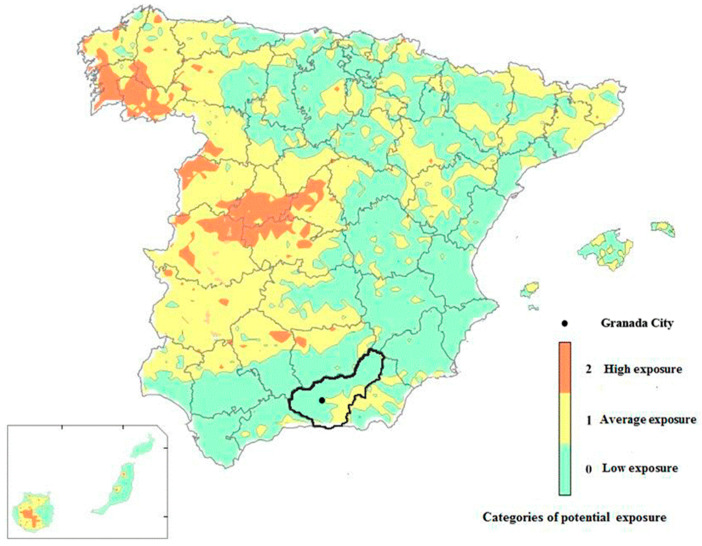
Map of exposure to radon-CSN (Spanish Nuclear Safety Council). The province of Granada is demarcated with a black line, and the capital (Granada) is indicated with a black dot. http://www.csn.es/en/mapa-de-exposicion-al-radon-en-espana (accessed on 22 April 2019).

National surveys and other investigations have described the range and the distribution of indoor radon levels in many European countries [10,24], observing mean values ranging from 20 to 140 Bq/m^3^ and geometric means ranging from 25 to 110 Bq/m^3^ [24]. García-Talavera et al. (2013) found that the areas most exposed to radon in Spain are in northwestern and western parts of the country, with a lesser exposure in southern regions (Figure 4). However, other regions are not classified as either radon-prone or non-prone because of inadequate sample sizes, as in the case of Andalusia, the setting of the present study [6]. A case-control study in Galicia, a radon-prone area of northwest Spain, found radon levels above 148 Bq/m^3^ in 21.3% of the homes and above 200 Bq/m^3^ in 12% [25]. A study of indoor concentrations using etched track dosimeters described a geometric mean concentration of (285 ± 2.5) Bq/m^3^ in Santiago de Compostela, a city in the same region; the authors observed that radon concentrations were higher during the night and 2.3-fold higher during holidays than working days, but they found no seasonal variations in concentrations [26]. Another study of 600 dwellings in Galicia described radon levels exceeding 200 Bq/m^3^ in 7.6%, 21.9%, and 68.5% of houses in areas of low, medium, and high radiation exposure, respectively [27]. High concentrations of indoor radon have also been found in workplaces in regions that are not characterized as radon-prone [12].

Other studies have revealed the influence of geological factors on radon risk in dwellings in northern Portugal. Winter measurements showed that average indoor radon concentrations were 85, 220, and 430 Bq/m^3^ in dwellings built on metasediments, AT1 granite, and AT2 granite, respectively [28]. Winter measurements by the same authors in a biotite granite region showed levels over 400 Bq/m^3^ in 62.6% of studied dwellings [29]. A cross-sectional study in a hydrothermal area (Furnas volcano, Azores, Portugal) found a mean indoor radon concentration of 115 Bq/m^3^, above the limit recommended by the WHO (100 Bq/m^3^) [1]. This was attributed by the authors to the lack of ventilation, exacerbated by the fact that measurements were made in the winter [30,31]. Among non-European countries, radon concentrations above the reference level were recorded in 51% of dwellings studied in bauxite-bearing areas of southern Adamawa in Cameroon [32]. In an investigation in a rural region of Southern Serbia, Žunić et al. reported mean indoor radon levels ranging between 39 and 202 Bq/m^3^ in schools and between 42 and 101 Bq/m^3^ in dwellings [33]. Mean findings were generally lower in the present study, although they were higher than might have been expected given the geology of the area and previous findings of lower levels in Southern Spain (Figure 1).

This study was not designed to evaluate the impact of indoor radon exposure on the health of staff in the workplaces under study. Nevertheless, various authors have proposed a link between this type of exposure and the risk of lung cancer [17,18]. Barros-Dios et al. found a more than two-fold increase in the risk of lung cancer at radon exposure levels above 37 Bq/m^3^ [25]. However, it is not yet clear whether radon exposure causes diseases other than lung cancer. A study of 28,557 participants in South Korea found that significantly higher indoor radon exposure was experienced by those who suffered from a stroke, which was more frequent with increased indoor radon levels (*p* < 0.001). The authors reported that indoor radon levels increased the risk of stroke after adjusting for potential confounding factors (OR: 1.004 (95 CI: 1.001–1.007), *p* = 0.010), and they observed an association between indoor radon exposure over 100 Bq/m^3^ and this disease (OR: 1.242 (95 CI: 1.069–1.444), *p* = 0.005) [30]. A prospective trial in the USA found a significant association between radon exposure and mortality from chronic obstructive pulmonary disease (COPD) [34]. However, the relationship of this exposure to diseases other than lung cancer, especially leukemia and COPD, remains controversial [35,36,37,38]. The United Nations Scientific Committee on the Effects of Atomic Radiation (UNSCEAR) and the International Commission on Radiological Protection (ICRP) described an increase of 0.16 in excess relative risk per 100 Bq/m^3^ [36]. Levels above 148 Bq/m^3^ were described as requiring action by the US Environmental Protection Agency (USEPA) [39] in 2012, and levels above 100 Bq/m^3^ were specified by the WHO [1]. A European Directive in 2013 set an annual exposure limit of 300 Bq/m^3^ for housing and closed spaces [22]. There has been a demand for strong regulations to avoid this exposure, given that an extremely small amount of radon in the air can be highly hazardous to health [14]. Given variations among published protocols or guidelines for measuring radon [40,41,42], there appears to be a need for a national plan to gather the endemic characteristics of each region in relation to its geological features, meteorological conditions, and the types of construction and materials used. Indoor radon exposure levels are not usually taken into account by clinicians. In fact, no lung cancer risk score (predicting incidence or mortality) includes radon as a variable. Further studies are needed to find out the molecular pathways of radon that cause lung cancer and to explore the involvement of this radioactive gas in the development of other diseases. It is necessary to increase awareness among administrations, health professionals, and the general population about the risks of exposure, especially in radon-prone areas [17]. 

## 5. Conclusions

Among its 17 Sustainable Development Goals, the United Nations called for more indoor radon measurements to estimate the exposure of general populations to this radioactive gas and evaluate its health effects. It is important for a National Radon Plan to be developed in Spain as in the majority of European countries. In the meantime, we consider that the population should be informed of the health risks associated with exposure to radon and its possible accumulation in indoor spaces, describing the factors that influence its concentration. These include type of exposure source, geographic localization, age of buildings, height of floor above ground, insulation level, and ventilation practices, among others. In the present study, ventilation appeared as an important protective factor, although only limited data were gathered on this variable. Because this gas is odorless, surveys of this type are essential to detect and monitor its presence and ensure that workers are not exposed to this hazard, for which there is no zero-risk level. Reductions in radon exposure can be expected with the implementation of energy-saving policies for complete thermal insulation using appropriate construction materials and improving ventilation measures. A more comprehensive study is needed to verify and expand the present findings in order to obtain annual average values in accordance with Instruction IS-33 [43] on workplace exposure to radon. 

## Figures and Tables

**Table 1:** Technical data (Radon-Scout PLUS Radon monitor, SARAD GmbH, Germany).

Measurement Range	0 … 10 MBq/m^3^
Error	±5% within the whole range or smaller
	1.8 cpm/1000 Bq/m^3^ (regardless of the humidity)
Sensitivity	200 Bq/m^3^ with statistical error of 20% (1 σ) at 1 h interval
	1000 Bq/m^3^ with statistical error <10% (1 σ) at 1 h interval
	100 Bq/m^3^ with statistical error of 17% (1 σ) at 3 h interval
Response Time	120 min to 95% of final value
Internal Sensors:	
Relative humidity	(0 … 100%)
Temperature	(−20 … 40 °C)
Barometric pressure	(800 … 1200 mbar)
Battery operation	>90 days
Uncertainty	1–2 Bq/m^3^

**Table 2:** Radon concentrations.

University Building					Radon (Bq/m^3^)						T (°C)	% RH	P (mbar)	Radon
	Study Area	Floor	Sample Time (Hours)	Data Recorded	AM	SD	P50	P90	Min	Max	AM	AM	AM	Total Exposure (Bqh/m^3^)
School of Sciences	NV-warehouse	basement	151	52	37	21	29	70	9	88	17	45	947	5629
	V-warehouse	basement	162	54	12	8	12	23	0	38	14	62	948	1958
	Central laboratory	basement	1104	368	70	59	47	173	0	265	15	54	0	76,788
	Maintenance room	basement	42	14	40	27	29	69	9	109	17	45	947	1571
	Documentation-archives room	basement	214	214	32	21	26	62	0	97	20	44	951	6890
Royal Hospital	Porter’s office	ground	984	984	17	17	9	36	0	115	20	36	951	16,957
	Reading-study room	ground	842	842	95	46	97	150	0	247	22	47	942	80,293
School of Computer Sciences	Porter’s office	ground	360	120	14	8	12	24	0	39	23	38	943	4793

NV: non-ventilated; V: ventilated; AM: arithmetic mean; SD: standard deviation; P50: percentile 50; P90: percentile 90; Min: minimum; Max: maximum; T: temperature; RH: relative humidity; P: pressure.

## Data Availability

Not applicable.
